# Potential new targets for drug development in severe asthma

**DOI:** 10.1186/s40413-018-0208-1

**Published:** 2018-10-25

**Authors:** Linda Zhu, Christina E. Ciaccio, Thomas B. Casale

**Affiliations:** 1 Department of Internal Medicine, The University of Chicago, Chicago, IL USA; 2 Department of Internal Medicine, NorthShore Health System, Chicago, IL USA; 3 Department of Pediatrics, The University of Chicago, 5841 South Maryland Avenue MC 5042, Chicago, IL 60637 USA; 4 Department of Medicine, The University of South Florida, Tampa, Florida, USA

**Keywords:** Asthma, Severe asthma, Asthma therapeutics, Drug targets, Biologics

## Abstract

In recent years there has been increasing recognition of varying asthma phenotypes that impact treatment response. This has led to the development of biological therapies targeting specific immune cells and cytokines in the inflammatory cascade. Currently, there are two primary asthma phenotypes, Type 2 hi and Type 2 lo, which are defined by eosinophilic and neutrophilic/pauci- granulocytic pattern of inflammation respectively. Most biologics focus on Type 2 hi asthma, including all four biologics approved for treatment of uncontrolled asthma in the United States — omalizumab, mepolizumab, reslizumab, and benralizumab. Potential new targets for drug development are being investigated, such as IL-13, IL-4α receptor, CRTH2, TSLP, IL-25, IL-13, IL-17A receptor, and CXCR2/IL-8. This review will discuss the role of these molecules on the inflammatory response in uncontrolled asthma and the emerging biologics that address them. Through the delineation of distinct immunological mechanisms in severe asthma, targeted biologics are promising new therapies that have the potential to improve asthma control and quality of life.

## Background

Asthma is a chronic disorder of the airways characterized by inflammation, reversible airflow obstruction and bronchial hyperresponsiveness, which is an increased sensitivity of the airways to a variety of stimuli resulting in bronchoconstriction [1]. Because underlying inflammation is central to the disease process, the mainstays of asthma therapy include inhaled corticosteroids (ICS) and systemic corticosteroids to prevent and treat exacerbations and to decrease symptoms. In recent years, there has been increasing recognition of patients whose asthma control is refractory to steroids, which has led to the delineation of contrasting asthma phenotypes. Different phenotypes have varying pathogenic pathways of inflammation, resulting in varying intensity of disease and therapeutic response to standard therapy. Currently, two major asthma phenotypes, Type 2 hi (T2-hi) and Type 2 lo (T2-lo), have been identified [2, 3].

T2-hi asthma is characterized by eosinophilic inflammation. In this pathway, airway epithelial cells and inflammatory cells such as mast cells, T-helper type 2 cells (Th2), type 2 innate lymphoid cells (ILC-2) release cytokines and mediators including IL-4, IL-5, IL-13, IgE, and thymic stromal lymphopoietin (TSLP) to induce airway inflammation [2, 4]. Several biomarkers have been used to identify these patients. High blood and sputum eosinophils levels, fractional exhaled nitric oxide (FeNO), periostin, and dipeptidyl pepdidase-4 (DPP-4) levels have been shown to correlate with a Th2 inflammatory response [5]. Since these biomarkers can be measured and often predict responsiveness to corticosteroids and T2 blockers, the majority of the biological agents developed target mediators of the T2-hi asthma profile.

**Fig. 1:**
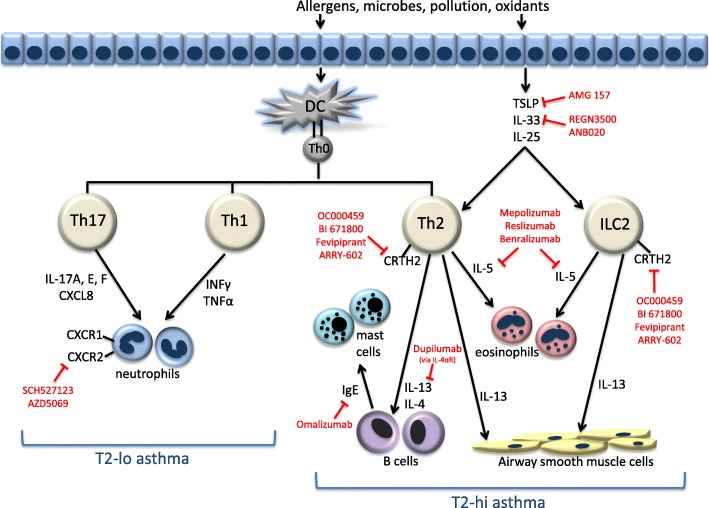
Pathophysiological mechanisms of T2-hi and T2-lo asthma and the current biologics that target them

T2-lo asthma (also classified as Th1-high or Th1/Th7-high) is characterized by a neutrophilic or pauci-granulocytic pattern of inflammation. Mediators of neutrophilic pathway include IL-8, IL-17, IL-23, which are important cytokines for neutrophil growth, differentiation and chemotaxis [2–4]. Corticosteroids are less effective in T2-lo asthma compared to T2-hi. Because few biomarkers have arisen to define this phenotype, determining the patient population that will respond to biologics targeting neutrophilic inflammation has been difficult (Fig. 1) [4, 5].

## Established biological agents

Currently, the FDA has approved omalizumab, mepolizumab, reslizumab, and benralizumab for the treatment of uncontrolled asthma (Table 1). Omalizumab is a humanized monoclonal antibody to IgE that blocks IgE interaction to high affinity receptor FcεRI on mast cells and other inflammatory cells. It is more efficacious in individuals with higher levels of blood eosinophils, FeNO or blood periostin. Treatment with omalizumab for 48 weeks demonstrated a greater percentage reduction of exacerbations in patients with high FeNO levels (≥19.5 ppb) compared to low FeNO levels (< 19.5 ppb) (53%; 95% CI 37–70; *P* = 0.001 vs. 16%; 95% CI -32 to 46; *P* = 0.45), high baseline eosinophil counts (≥260/μL) compared to low eosinophil counts (< 260 μL) (32%; 95% CI 11–48; P 0.005 vs. 9%; 95% CI -24 to 34; *P* = 0.54) and high periostin levels (≥ 50 ng/mL) compared to low periostin levels (< 50 ng/mL) (30%; 95% CI -2 to 51; *P* = 0.07) vs. 3%; 95% CI -43 to 32, *P* = 0.94) [6]. Similarly, patients with high eosinophil count (≥ 300/μL) had decreased rate of exacerbations versus placebo (0.25 vs. 0.59; RR 0.41; 95% CI 0.20–0.82) and patients with low eosinophils counts (< 300/μL) showed no improvement (0.17 vs. 0.16; RR 1.07; 95% CI 0.45–2.53) [7]. In two phase 3 clinical trials, patient receiving omalizumab had a relative exacerbation rate reduction of 55% compared to placebo (95% CI 32–70%; *P* = 0.002), and this effect was more notable with higher eosinophil counts: ≥ 200/μL, 55% mean exacerbation rate reduction (95% CI 25–75%; *P* = 0.002); ≥ 300/μL, 67% rate reduction (95% CI 36–84%; *P* = 0.001); ≥ 400/μL, 74% rate reduction rate (95% CI 40–88%; *P* = 0.001) [8]. Omalizumab is also the only biological agent approved thus far for pediatric patients (ages 6 and above) in the USA, where it has been shown to reduce free IgE levels, decrease the frequency of asthma exacerbations, and improve quality of life [9].

**Table 1:** FDA approved therapies

	Mechanism of Action	Biomarker	Outcomes	Significant Adverse Events	References
Omalizumab	Blocks IgE interaction to FcεRI	↑FeNO (> 19.5 ppb) ↑Peripheral eosinophils (≥200/uL)	Decreased exacerbations Reduced IgE levels Improved quality of life	Cardiovascul ar and cerebrovascu lar event risk	Hanania 2013 [6] Busse 2013 [7] Casale 2017 [4] Chipps 2017 [9]
Mepolizumab	Anti-IL5	↑Peripheral eosinophils (> 150 or 300/uL) or sputum eosinophilia (> 3%)	Decreased exacerbations Reduced prednisone dose Decreased blood and sputum eosinophils Improved quality of life	Herpes Zoster; Helminth infections	Nair 2009 [10] Pavord 2012 [11] Liu 2013 [14] Ortega 2014 [13] Bel 2014 [12]
Reslizumab	Anti-IL5	↑Peripheral eosinophils (> 400/uL) or sputum eosinophilia (> 3%)	Decreased exacerbations Decreased blood and sputum eosinophils Improved FEV_1_	Helminth infections; CPK elevation	Castro 2011 [15] Castro 2015 [16] Corren 2016 [17]
Benralizumab	Anti-IL5Ralpha	↑Eosinophils ↑FeNO	Decreased exacerbations (higher eosinophilia more predictive of response) Improved FEV_1_	Helminth infections	Castro 2014 [18] FitzGerald 2016 [19] Bleecker 2016 [20]

Mepolizumab and reslizumab are monoclonal antibodies to IL-5. Mepolizumab has been shown to increase the median time to exacerbation (20 weeks vs. 12 weeks; *P* = 0.003), reduce prednisone use (83.8 ± 33.4% vs. 47.7 ± 40.5%; *P* = 004), and decrease the number of sputum and blood eosinophils within normal limits (*P* = 0.005 and *P* = 0.004 respectively) [10]. The DREAM study further demonstrated that mepolizumab reduced the number of exacerbations: 48% at a dose of 75 mg (95% CI 31–61%; *P* < 0.0001); 39% at 250 mg (CI 95% 19–54%; *P* = 0.0005); 52% at 750 mg (95% CI 36–64%; *P* < 0.0001) and blood and sputum eosinophils [11–13]. The SIRIUS study confirmed that mepolizumab reduces glucocorticoid use (OR 2.39; 95% CI 1.25–4.56; *P* = 0.008) [12]. In a meta-analysis of randomized placebo-controlled trials, mepolizumab was also found to improve Asthma Quality of Life Questionnaire scores (MD 0.26; 95% CI 0.03–0.49, *P* = 0.03) in patients with eosinophilic asthma, but no improvement in forced expiratory volume in 1 s (FEV_1_) was observed [13, 14]. Mepolizumab has recently been approved in Europe for children down to 6 years of age.

Similarly, reslizumab reduced sputum eosinophils (median percentage reduction 95.4% in reslizumab group vs. 38.7% in placebo; *P* = 0.0068) [15], improved FEV_1_ (change from baseline 0.18 in reslizumab group vs. -0.08 in placebo; *P* = 0.0023) [15] and reduced frequency of asthma exacerbations (study 1- RR 0.50; 95% CI 0.37–0.67; *P* < 0.0001; study 2- RR 0.41; 95% CI 0.28–0.59; *P* < 0.0001) [15, 16]. No significant effects on symptom control or lung function was seen in patients with poorly controlled asthma without stratifying for high eosinophils counts [17].

Benralizumab is a monoclonal IL-5 receptor antagonist that acts by binding to the α-chain of the IL-5 receptor on eosinophils and basophils to induce apoptosis via antibody-dependent cell-mediated cytotoxicity. Phase 2b clinical trials showed benralizumab 100 mg decreased exacerbations in patients with uncontrolled asthma and high baseline blood eosinophils (0.34 vs. 0.57; reduction 41%; 80% CI 11–60; *P* = 0.096), but not at lower doses of 20 mg and 100 mg [18]. In recently completed Phase 3 clinical trials, benralizumab demonstrated reduction in annual asthma exacerbations and improved pre-bronchodilator FEV_1_ in patients with severe asthma uncontrolled by high-dosage ICS plus long acting beta-adrenoceptor agonist (LABA) with blood eosinophils 300/μL or greater [19, 20]. In patients with lower blood eosinophils, improvements in both exacerbation rates and FEV_1_ were noted but these tended to not be clinically or statistically significant. A small study in patients presenting to ER with acute asthma also showed that one dose of benralizumab reduced rate and severity of asthma exacerbations by 50% over 12 weeks [21].

## Novel targets in T2-hi asthma

### Interleukin-4α receptor

IL-4α receptor is the common receptor domain for both IL-13 and IL-4. Dupilumab is a fully humanized monoclonal antibody to the alpha chain of the IL-4α receptor (Table 2). In patients with moderate to severe asthma with elevated serum eosinophils (≥300/μL) or sputum eosinophils (≥3%), dupilumab decreased frequency of asthma exacerbations when ICS and LABA were withdrawn by 87% (OR 0.08; 95% CI 0.02–0.28; *P* < 0.001), improved FEV_1_ and Asthma Control Questionnaire scores and decreased Th2 associated inflammatory markers [22]. Dupilumab has also recently been shown to be effective in reducing pulmonary function and asthma exacerbation frequency regardless of baseline blood eosinophil levels, although greater improvements were noted in patients with higher blood eosinophil levels. In the subgroup of patients with eosinophilia ≥300/μL, all doses of dupilumab except for 200 mg every 4 weeks increased FEV_1_ significantly (300mg every 4 weeks, *P*= 0.0212; 200mg every 2 weeks, *P*=0.0008; 300mg every 2 weeks, *P*=0.0063), and similar improvements were also noted in the subgroup with eosinophila <300 μL (200 mg every 2 weeks, *P* = 0.0057; 300 mg every 2 weeks, *P* = 0.0262) and overall population (200 mg every 2 weeks, *P* < 0.001; 300 mg every 2 weeks, *P* = 0.002) with dupilumab every 2 weeks [23]. In Phase 3 studies, dupilumab 200 mg every 2 weeks reduced annualized rate of severe asthma exacerbations by 47.7% compared to placebo (*P* < 0.001) and increased FEV_1_ by 0.32 L (difference vs. match placebo, 0.14 L; *P* < 0.001), and greater benefits were again seen in patients with blood eosinophilia ≥300/μL [24]. In patient with glucocorticoid-dependent severe asthma, dupilumab also reduced glucocorticoid use: − 70.1% in dupilumab group vs. -41.9% in placebo group (*P* < 0.001) [25].

**Table 2:** Awaiting FDA approval

	Mechanism of Action	Biomarker	Outcomes	Significant Adverse Events	References
Dupilumab (Completed phase III, under FDA review)	Anti-IL4R	↑Peripheral eosinophils (≥300/uL) ↑Sputum eosinophils (≥3%)	Decreased exacerbations and symptoms Improved FEV_1_ Decreased glucocorticoid use Decreased FeNO		

It is currently awaiting FDA approval for use in asthma. Additionally, it is under investigation for the treatment of severe asthma in children and has recently been approved for severe atopic dermatitis in patients over 18 years of age.

### Interleukin-13

IL-13 mediates inflammation through proliferation of bronchial fibroblasts, recruitment of eosinophils and basophils, and IgE synthesis [26]. Periostin and DPP-4 are produced by bronchial fibroblasts and epithelial cells via IL-13, and thus have been used as biomarkers of this pathway. Two monoclonal antibodies to IL-13, lebrikizumab and tralokinumab, have been studied in clinical trials (Table 3). Lebrikizumab initially demonstrated improved FEV_1_ in uncontrolled asthma, particularly in the high-periostin subgroup [27, 28]. However, phase 3 trials clinical trial did not show significant reduction in asthma exacerbation, even in a biomarker-rich population, leading to the discontinuation of this drug’s development [28]. Tralokinumab also did not significantly reduce asthma exacerbation rates or improve lung function in patients with severe uncontrolled asthma, except in a post hoc analysis of subgroups with higher periostin or DPP-4 levels [29]. Recent studies did not show clinically meaningful improvements and further development of tralokinumab for asthma has ceased.

**Table 3:** In clinical trials/Novel Targets

Specific target	Drug	Biomarkers	Outcomes	Potential Adverse Events	References
CRTH2	OC000459 (Phase 2)	None	Improved FEV1 and quality of life scores	Cardiovascular and cerebrovascular events; helminthic and viral infections	Barnes 2012 [31] Pettipher 2014 [32]
	BI 671800 (Phase 2)	None	Mixed results for FEV_1_ improvement Mixed results for asthma control scores	Cardiovascular and cerebrovascular events; helminthic and viral infections	
	QAW039/Fevipiprant (Phase 3)	↑Sputum eosinophils (≥2% for Gonem et al.)	Decreased sputum eosinophils Mixed results for FEV_1_ improvement Mixed results for asthma control scores	Cardiovascular and cerebrovascular events; helminthic and viral infections	Gonem 2016 [39] Erpenbeck 2016 [37] Bateman 2017 [38]
	ARRY-602 (Phase 2)	↑Th2 associated biomarkers	Improved FEV1, asthma control and quality of life score	Cardiovascular and cerebrovascular events; helminthic and viral infections	Wenzel 2014
TSLP	AMG 157/Tezepelumab (Phase 2)	None	Reduced early and late response (FEV1 decrease) to allergen challenge Decreased exacerbations Improved FEV1 Decreased blood eosinophils, FeNO, and total IgE	Cardiovascular and cerebrovascular events; skin infections; Guillain-Barre	Gauvreau 2014 [48] Corren 2017 [49]
CXCR2/IL-8	SCH527123/Navarixin (Phase 2)	↑Sputum neutrophils (> 40%)	Decreased sputum neutrophils Trend towards improved asthma control	Nasopharyngitis; neutropenia	Nair 2012 [73]
	AZD5069 (Phase 2)	↑Blood neutrophils (≥ 2.7 × 109/L)	No difference in exacerbations	Nasopharyngitis; neutropenia	O’Byrne 2016 [74]
IL-33	REGN3500 (Phase 1) ANB020 (Phase 2)	Results pending		Cardiovascular and cerebrovascular events; helminthic and viral infections	
IL-25		No biologics in human trials yet		Cardiovascular and cerebrovascular events; helminthic and viral infections	
IL-17A	CCJM112 (Phase 2)	Low IgE and Blood Eosinophils			

### Chemoattractant receptor-homologous molecule expressed on Th2 cells (CRTH2)

CRTH2 (DP2) is a G protein-coupled receptor expressed on key immune cells, specifically Th2 cells, ILC-2, eosinophils and basophils. Activated mast cells secrete prostaglandin D_2_ (PGD_2_), which binds CRTH2 to promote release of type 2 cytokines IL-4, IL-5, IL-13 from ILC-2 and Th2 cells in addition to stimulating eosinophilic chemotaxis and degranulation [30]. Several studies on CRTH2 antagonists, including OC000459, QAW039 (fevipiprant), ARRY-602, AMG853, and setipiprant, have demonstrated mixed results. In a randomized controlled trial, the mean improvement in FEV_1_ was 9.2% for patients on treatment with OC000459 (200 mg twice daily) versus 1.8% placebo (*P* = 0.037) in the per protocol population, which excluded non-compliant subjects [31–33]. There was also a small but significant improvement in the Standardized Asthma quality of life questionnaire (difference from placebo = 0.37, *P* = 0.0022) and night-time symptom scores (mean reduction of 0.37 vs. 0.12, *P* = 0.022) [31–33]. OC00459 at 3 different doses (25 mg once daily, 200 mg once daily or 100 mg twice daily) improved FEV_1_ by 95 mL in the pooled dose group compared to placebo (*P* = 0.024) [32]. In the post hoc analysis of patients with blood eosinophils ≥250/μL, the eosinophilic subgroup showed greater improvement in FEV_1_ (mean increase of 220 mL compared to placebo; *P* = 0.005), particularly in a younger population (mean increase of 355 mL in subjects ≤40 years old compared to placebo; *P* = 0.007) [32]. Similarly, ARRY-502 showed slightly improved FEV_1_ (3.9%; *P* = 0.02), Asthma Control Questionaaire-7 (*P* < 0.001), beta-agonist use (*P* < 0.001), and symptom free days (*P* = 0.07) compared to placebo in patients with elevated Th2 associated biomarkers [34]. BI 671800 has varying results, ranging from no significant difference to small significant improvement in FEV_1_ and/or asthma control scores depending on the study [35, 36]. Results for QAW039 (fevipiprant) are also mixed. In a phase II study, no difference in FEV_1_ or Asthma Control Questionnaire was observed in patients with mild to moderate uncontrolled asthma, but a subgroup analysis suggested improvement of both end points in patients with a FEV_1_ < 70% of predicted at baseline (FEV_1_ change of 207 mL between QAW039 and placebo; 90% CI 96–319; *P* = 0.002; Asthma Questionnaire Score change of − 0.41; 90% CI -0.69 to − 0.13; *P* = 0.009) [37]. A subsequent study showed a small improvement in FEV_1_ in asthmatics who were uncontrolled on low dose ICS (maximally model-averaged difference of 0.112 L compared to placebo, *P* = 0.0035) though similar results were observed with montelukast [38]. In patients with elevated sputum eosinophils (≥2%), mean eosinophil percentage was reduced by 4.5 times baseline in the fevipiprant treatment group compared to 1.3 times baseline in the placebo (difference between groups 3.5 times; 95% CI 1.7–7.0; *P* = 0.0014) [39]. AMG853 and setipiprant have been discontinued due to poor efficacy [40, 41]. Of note, the studies that stratified asthma by phenotype found greater efficacy in subgroups with elevated serum eosinophils [32, 39] and FeNO [34], which may explain why other trials with CRTH2 antagonists that did not stratify by T2 inflammation did not yield positive results.

### Thymic stromal Lymphopoietin (TSLP)

Airway epithelial derived cytokines such as TSLP, IL-25, and IL-33 drive allergic inflammatory responses to airway damage. TSLP acts on dendritic cells, mast cells, ILC-2 cells, and eosinophils to promote Th2 cell differentiation and secretion of cytokines such as IL-4, IL-5, and IL-13 [42–44]. There is an increased expression of TSLP messenger RNA and proteins in the airways of asthmatic patients compared to controls, and the degree of expression correlated with severity of asthma and lung function [45, 46]. Additionally, polymorphisms in the TSLP gene are associated with both childhood atopic and adult asthma, possibly via higher TSLP production in response to viral respiratory infections [47]. Currently, the only therapeutic targeting TSLP is AMG 157, a human anti-TSLP monoclonal antibody that binds to TSLP to block interaction with its receptor. Compared to placebo, AMG 157 reduced the maximal percentage decrease in FEV_1_ by 34.0% during the late response to allergen challenge on day 42 (*P* = 0.09) and 45.9% smaller on day 84 (*P* = 0.02) in patients with stable allergic asthma [48]. It also attenuated measures of airway inflammation, including FeNO levels and eosinophils counts in blood and sputum [48]. Recently, in a phase 2 trial, AMG 157 (tezepelumab) reduced exacerbation rates (by 61% with dose of 70 mg every 4 weeks compared to placebo rate, *P* < 0.0001; by 71% with 210 mg every 4 weeks, *P* < 0.0001; by 66% with 280 mg every 2 weeks, *P* < 0.0001), increased prebronchodilator FEV_1_ (difference of 0.12 L with 70 mg every 4 weeks compared to placebo, *P* = 0.01; 0.11 L with 210 mg every 4 weeks, *P* = 0.02; 0.15 L with 280 mg every 2 weeks, *P* = 0.002) and decreased Th2 markers of inflammation in patients with uncontrolled asthma despite LABA combined with medium or high dose ICS [49]. Interestingly, the decrease in the annualized asthma exacerbation rate was observed regardless of baseline blood eosinophil count, suggestive that tezepelumab may be effective in asthmatics without an eosinophilic inflammation profile [49].

### Interleukin-25

IL-25 (also known as IL-17E) is an epithelial derived alarmin that is a member of the IL-17 cytokine family. It is constitutively produced in bronchial epithelial cells and released on exposure to proteases, such as allergen proteases on dust mite extract, to activate a Th2 response [50]. IL-25 binds to the IL-25 receptor, which is composed of IL-17RA and IL-17RB, to potentiate eosinophilia as well as production of Th2 cytokines IL-4, IL-5, IL-13 [51]. Patient with allergic asthma had significantly higher expression of IL-17RB and IL-17RA on eosinophils compared to atopic non-asthmatics and normal controls subjects, and higher serum IL-25 levels compared to control [52]. Additionally, asthmatic patients with higher levels of IL-25 messenger RNA levels had greater airway hyperresponsiveness to allergens, increased serum IgE, airway and blood eosinophils levels and more beneficial responses to inhaled corticosteroids compared asthmatics with low IL-25 expression [53]. IL-25 also enhanced smooth muscle contractility of bronchial rings from asthmatic donors in the setting of methacholine induction [54]. There is currently no biologics against IL-25 directly, however brodalumab (discussed separately in IL-17 section) indirectly blocks IL-25 activity by binding to IL-17RA.

### Interleukin-33

IL-33 is a member of the IL-1 cytokine family that is produced by airway epithelial cells, smooth muscle cells and endothelium and expressed on immune cells such as dendritic cells, macrophages and mast cells. IL-33 binds to IL-1R1 and IL-1RAcP receptor complex to activate production of various cytokines, including type 2 cytokines IL-4, IL-5, and IL-13 [55]. In particular, IL-33 activates ILC-2, which promote persistence of airway eosinophilia in patients with severe asthma refractory to steroids via production of IL-5 and IL-13 [56]. In animal studies, IL-33 knockout mice did not develop lung eosinophilia after allergen induction, which suggests that IL-33 is essential for eosinophilic infiltration [57]. In a murine study, intradermal administration of ovalbumin in setting of excess IL-33 promoted antigen-induced allergic airway inflammation [58]. Furthermore, anti-IL33 antibody inhibited Th2 cytokine production, airway inflammation/remodeling, and mucous hypersecretion in mice [59].

Patients with severe asthma have higher mRNA expression of IL-33 on lung tissue compared to controls [60]. In a metaanalysis, children with asthma were found to have a higher serum level of IL-33 compared to healthy children, though was significant heterogeneity amongst studies [61]. These data suggest that IL-33 may be a key regulator, inflammatory marker, and potential biomarker of severe, refractory asthma. Currently, there are two anti-IL33 antibodies, REGN3500 and ANB020, being investigated with results pending.

## Novel targets in T2-lo asthma

### Interleukin-17A

IL-17A and IL-17F are members of the IL-17 cytokine family. Produced by Th17 cells, they act on epithelial cells to potentiate cytokines that induce local recruitment of neutrophils [62]. In patients with asthma, elevated levels of IL-17A and IL-17F are found in bronchoalveolar lavage fluid and airway tissue and positively correlate with disease severity and neutrophil inflammation [63–65]. IL-17A also acts on airway smooth muscle cells to mediate airway hyperresponsiveness. In studies with mice models and human bronchial tissue, IL-17A and IL-25 have been found to enhance methacholine-induced contractile force generation of airway smooth muscle [54, 66].

IL-17A and IL17F bind to receptor complexes that have IL-17RA as the common subunit. Brodalumab is a human monoclonal antibody that binds to IL-17RA, thereby blocking activity of IL-17A, IL-17B, and IL-25 (Table 3). In a randomized control trial of patients with uncontrolled moderate to severe asthma on inhaled corticosteroids, brodalumab did not demonstrate a difference Asthma Control Questionnaire score, lung function or asthma symptoms in the overall study population, but did show a nominal significance in asthma control scores (estimated treatment difference of 0.53; *P* = 0.02) in a small subgroup with high bronchodilator reversibility (post bronchodilator FEV_1_ improvement ≥20%) at a dose of 210 mg [67]. However, this drug led to mental health issues including suicide in clinical trials which resulted in discontinuation of further development for asthma. Secukinumab, an IL-17A blocker, did not show a significant difference in total number of sputum neutrophils from baseline in healthy volunteers who developed acute neutrophilic airway inflammation following an ozone challenge. [68]. Preliminary data in a phase 2 study of AIN457 (secukinumab) in patients with uncontrolled asthma did not show a difference in Asthma Control Questionnaire scores, and has been terminated [69]. CCJM112, an anti-IL17A, is now in a phase 2 clinical trial for patients with low IgE and blood eosinophils.

### C-X-C motif chemokine receptor 2 (CXCR2)/Interleukin-8

IL-8 is a potent chemoattractant that mediates activation and migration of neutrophils to the sites of inflammation via the high affinity CXCR2 receptor. Increased sputum levels of IL-8 often preceded asthma exacerbations in severe asthmatics, and sputum IL-8 levels also correlated with development of late phase allergic airflow obstruction in atopic patients [70, 71]. IL-8 is elevated in the serum as well, but its role as a biomarker for disease activity remains controversial. In animal studies, a selective antagonist to CXCR2 and CXCR1 was found to suppress pulmonary neutrophilia and airway inflammation [72]. In a phase 2 study of patients with moderate to severe asthma with high neutrophils counts at baseline (> 40%), CXCR2 antagonist SCH527123 reduced sputum neutrophils counts by 36.3% compared to a 6.7% increase in the placebo group (*P* = 0.03), but only a trend towards improved asthma control was observed [73]. A subsequent randomized clinical trial using CXCR2 antagonist AZD5069 as add on therapy for patient with severe asthma demonstrated no significant difference in frequency of exacerbations though it was limited by the overall low exacerbation rate [74]. Additionally, there currently is no validated marker for neutrophilic airway inflammation, thus the study population was not stratified by neutrophilic phenotype. While a subset of asthma (T2-lo) patients do have neutrophil predominance, a correlation between neutrophil reduction and clinical benefit remains to be seen.

## Conclusions

Asthma is a complex, heterogeneous disease with varying phenotypes that affect treatment response. The characterization of the T2-hi profile has led to the development of specific biological agents that target the immune cells and cytokines in the inflammatory cascade. Currently, all of the FDA approved biologics (omalizumab, mepolizumab, reslizumab, benralizumab) and the majority of potential therapeutic targets focus on this pathway. While some targeted therapies have had promising preliminary results, others have not shown a significant biological or clinical response due to several potential limitations. First, blocking a single cytokine or inflammatory cell may be insufficient to reduce inflammation, possibly because of compensatory response from a different cytokine. Studies with cytokine deficient mice have shown the importance of integrated signaling activity between IL-13, IL-4, and IL-5 [75]. Thus, targeting receptors that multiple cytokines converge upon may be more effective. For example, dupilumab inhibits IL-4α receptor (a common receptor domain for both IL-13 and IL-4) and has demonstrated efficacy in Phase 3 trials [22, 23]. Secondly, identification of the appropriate study population is key in determining response to therapy. For instance, studies of CRTH2 antagonists that stratified by phenotype found greater response in subgroups with elevated serum eosinophils [32] and FeNO [34] whereas those that did not stratify by T2 biomarkers did not. This requires reliable and readily available biomarkers to define the asthma phenotype. At present, there is a need to establish biomarkers for T2-low asthma patients, who often do not respond to steroids and current biological agents, and to investigate possible differing endotypes even within the T2-low phenotype [5]. By delineating distinct immunological mechanisms in severe asthma, targeted biologics are promising new therapies that have the potential to improve asthma control and quality of life.

## Abbreviations

CI: Confidence interval
CRTH2 (DP2): Chemoattractant receptor-homologous molecule expressed on Th2 cells (prostaglandin D_2_ receptor)
CXCR2: C-X-C motif chemokine receptor 2
DPP-4: Dipeptidyl pepdidase-4
FeNO: Fractional exhaled nitric oxide
FEV_1_: Forced expiratory volume at 1 s
ICS: Inhaled corticosteroids
IL: Interleukin
ILC-2: Type 2 innate lymphoid cell
LABA: Long acting beta-adrenoceptor agonist
OR: Odds ratio
PGD_2_: Prostaglandin D_2_
T2-hi: Type 2 hi asthma
T2-lo: Type 2 lo asthma
Th2: T-helper type 2 cell
TLSP: Thymic stromal lymphopoietin

## References

[1] National Asthma Education and Prevention Program. Expert Panel Report 3 (EPR-3) (2007). Guidelines for the diagnosis and Management of Asthma-Summary Report 2007. J Allergy Clin Immunol.

[2] Stokes JR, Casale TB (2016). Characterization of asthma endotypes: implications for therapy. Ann Allergy Asthma Immunol.

[3] Wenzel SE (2012). Asthma phenotypes: the evolution from clinical to molecular approaches. Nat Med.

[4] Casale TB (2017). Biologics and biomarkers for asthma, urticaria, and nasal polyposis. J Allergy Clin Immunol.

[5] Berry A, Busse WW (2016). Biomarkers in asthmatic patients: has their time come to direct treatment?. J Allergy Clin Immunol.

[6] Hanania NA (2013). Exploring the effects of omalizumab in allergic asthma: an analysis of biomarkers in the EXTRA study. Am J Respir Crit Care Med.

[7] Busse W, Spector S, Rosén K, Wang Y, Alpan O (2013). High eosinophil count: a potential biomarker for assessing successful omalizumab treatment effects. J Allergy Clin Immunol.

[8] Casale T. B., Chipps B. E., Rosén K., Trzaskoma B., Haselkorn T., Omachi T. A., Greenberg S., Hanania N. A. (2017). Response to omalizumab using patient enrichment criteria from trials of novel biologics in asthma. Allergy.

[9] Chipps BE (2017). Omalizumab in children with uncontrolled allergic asthma: review of clinical trial and real-world experience. J Allergy Clin Immunol.

[10] Nair P (2009). Mepolizumab for prednisone-dependent asthma with sputum eosinophilia. N Engl J Med.

[11] Pavord ID (2012). Mepolizumab for severe eosinophilic asthma (DREAM): a multicentre, double-blind, placebo-controlled trial. Lancet.

[12] Bel EH (2014). Oral glucocorticoid-sparing effect of mepolizumab in eosinophilic asthma. N Engl J Med.

[13] Ortega HG (2014). Mepolizumab treatment in patients with severe eosinophilic asthma. N Engl J Med.

[14] Liu Y, Zhang S, Li D-W, Jiang S-J (2013). Efficacy of anti-interleukin-5 therapy with mepolizumab in patients with asthma: a meta-analysis of randomized placebo-controlled trials. PLoS One.

[15] Castro M (2011). Reslizumab for poorly controlled, eosinophilic asthma: a randomized, placebo-controlled study. Am J Respir Crit Care Med.

[16] Castro M (2015). Reslizumab for inadequately controlled asthma with elevated blood eosinophil counts: results from two multicentre, parallel, double-blind, randomised, placebo-controlled, phase 3 trials. Lancet Respir Med.

[17] Corren J, Weinstein S, Janka L, Zangrilli J, Garin M (2016). Phase 3 study of Reslizumab in patients with poorly controlled asthma: effects across a broad range of eosinophil counts. Chest.

[18] Castro M (2014). Benralizumab, an anti-interleukin 5 receptor α monoclonal antibody, versus placebo for uncontrolled eosinophilic asthma: a phase 2b randomised dose-ranging study. Lancet Respir Med.

[19] FitzGerald JM (2016). Benralizumab, an anti-interleukin-5 receptor α monoclonal antibody, as add-on treatment for patients with severe, uncontrolled, eosinophilic asthma (CALIMA): a randomised, double-blind, placebo-controlled phase 3 trial. Lancet.

[20] Bleecker ER (2016). Efficacy and safety of benralizumab for patients with severe asthma uncontrolled with high-dosage inhaled corticosteroids and long-acting β2-agonists (SIROCCO): a randomised, multicentre, placebo-controlled phase 3 trial. Lancet.

[21] Nowak RM (2015). A randomized trial of benralizumab, an antiinterleukin 5 receptor α monoclonal antibody, after acute asthma. Am J Emerg Med.

[22] Wenzel S (2013). Dupilumab in persistent asthma with elevated eosinophil levels. N Engl J Med.

[23] Wenzel S (2016). Dupilumab efficacy and safety in adults with uncontrolled persistent asthma despite use of medium-to-high-dose inhaled corticosteroids plus a long-acting β2 agonist: a randomised double-blind placebo-controlled pivotal phase 2b dose-ranging trial. Lancet.

[24] Castro M (2018). Dupilumab efficacy and safety in moderate-to-severe uncontrolled asthma. N Engl J Med.

[25] Rabe KF (2018). Efficacy and safety of Dupilumab in glucocorticoid-dependent severe asthma. N Engl J Med.

[26] Gallelli L, Busceti MT, Vatrella A, Maselli R, Pelaia G (2013). Update on anticytokine treatment for asthma. Biomed Res Int.

[27] Corren J (2011). Lebrikizumab treatment in adults with asthma. N Engl J Med.

[28] Hanania NA (2016). Efficacy and safety of lebrikizumab in patients with uncontrolled asthma (LAVOLTA I and LAVOLTA II): replicate, phase 3, randomised, double-blind, placebo-controlled trials. Lancet Respir Med.

[29] Brightling CE (2015). Efficacy and safety of tralokinumab in patients with severe uncontrolled asthma: a randomised, double-blind, placebo-controlled, phase 2b trial. Lancet Respir Med.

[30] Farne H, Jackson DJ, Johnston SL (2016). Are emerging PGD2 antagonists a promising therapy class for treating asthma?. Expert Opin Emerg Drugs.

[31] Barnes N (2012). A randomized, double-blind, placebo-controlled study of the CRTH2 antagonist OC000459 in moderate persistent asthma. Clin Exp Allergy.

[32] Pettipher R (2014). Heightened response of eosinophilic asthmatic patients to the CRTH2 antagonist OC000459. Allergy.

[33] Singh D (2013). Inhibition of the asthmatic allergen challenge response by the CRTH2 antagonist OC000459. Eur Respir J.

[34] Wenzel S, Hopkins R, Saunders M, Chantry D, Anderson L, Aitchison R, Eberhardt C, Bell S, Cole J, Wolfe JD, Spector S (2014). Safety and efficacy of ARRY-502, a potent, selective, Oral CRTh2 antagonist, in patients with mild to moderate Th2-driven asthma. J Allergy Clin Immunol.

[35] Hall IP (2015). Efficacy of BI 671800, an oral CRTH2 antagonist, in poorly controlled asthma as sole controller and in the presence of inhaled corticosteroid treatment. Pulm Pharmacol Ther.

[36] Miller D (2017). A randomized study of BI 671800, a CRTH2 antagonist, as add-on therapy in poorly controlled asthma. Allergy Asthma Proc.

[37] Erpenbeck VJ (2016). The oral CRTh2 antagonist QAW039 (fevipiprant): a phase II study in uncontrolled allergic asthma. Pulm Pharmacol Ther.

[38] Bateman ED (2017). Fevipiprant, an oral prostaglandin DP2 receptor (CRTh2) antagonist, in allergic asthma uncontrolled on low-dose inhaled corticosteroids. Eur Respir J.

[39] Gonem S (2016). Fevipiprant, a prostaglandin D2 receptor 2 antagonist, in patients with persistent eosinophilic asthma: a single-Centre, randomised, double-blind, parallel-group, placebo-controlled trial. Lancet Respir Med.

[40] Busse WW (2013). Safety and efficacy of the prostaglandin D2 receptor antagonist AMG 853 in asthmatic patients. J Allergy Clin Immunol.

[41] Diamant Z (2014). Setipiprant, a selective CRTH2 antagonist, reduces allergen-induced airway responses in allergic asthmatics. Clin Exp Allergy.

[42] Ziegler, AF. "The Biology of Thymic Stromal Lymphopoietin (TSLP)." Immunopharmacology. Ed. David R. Webb. San Diego: Elsevier; 2013. 129–156. 10.1016/B978-0-12-404717-4.00004-4 PMC4169878 23433457

[43] Allakhverdi Z (2007). Thymic stromal lymphopoietin is released by human epithelial cells in response to microbes, trauma, or inflammation and potently activates mast cells. J Exp Med.

[44] Soumelis V (2002). Human epithelial cells trigger dendritic cell mediated allergic inflammation by producing TSLP. Nat Immunol.

[45] Ying S (2008). Expression and cellular provenance of Thymic stromal Lymphopoietin and chemokines in patients with severe asthma and chronic obstructive pulmonary disease. J Immunol.

[46] Ying S (2005). Thymic stromal lymphopoietin expression is increased in asthmatic airways and correlates with expression of Th2-attracting chemokines and disease severity. J Immunol.

[47] Harada M (2011). Thymic stromal lymphopoietin gene promoter polymorphisms are associated with susceptibility to bronchial asthma. Am J Respir Cell Mol Biol.

[48] Gauvreau GM (2014). Effects of an anti-TSLP antibody on allergen-induced asthmatic responses. N Engl J Med.

[49] Corren J (2017). Tezepelumab in adults with uncontrolled asthma. N Engl J Med.

[50] Kouzaki H, Tojima I, Kita H, Shimizu T (2013). Transcription of interleukin-25 and extracellular release of the protein is regulated by allergen proteases in airway epithelial cells. Am J Respir Cell Mol Biol.

[51] Hurst SD (2002). New IL-17 family members promote Th1 or Th2 responses in the lung: in vivo function of the novel cytokine IL-25. J Immunol.

[52] Tang W (2014). IL-25 and IL-25 receptor expression on eosinophils from subjects with allergic asthma. Int Arch Allergy Immunol.

[53] Cheng D (2014). Epithelial interleukin-25 is a key mediator in Th2-high, corticosteroid-responsive asthma. Am J Respir Crit Care Med.

[54] Willis CR (2015). IL-17RA signaling in airway inflammation and bronchial Hyperreactivity in allergic asthma. Am J Respir Cell Mol Biol.

[55] Mitchell PD, O'Byrne PM (2017). Epithelial-derived cytokines in asthma. Chest.

[56] Smith SG (2016). Increased numbers of activated group 2 innate lymphoid cells in the airways of patients with severe asthma and persistent airway eosinophilia. J Allergy Clin Immunol.

[57] Kamijo S (2013). IL-33-mediated innate response and adaptive immune cells contribute to maximum responses of protease allergen-induced allergic airway inflammation. J Immunol.

[58] Han H, Ziegler SF. Intradermal administration of IL-33 induces allergic airway inflammation. Sci Rep. 2017;7(1706):1–8. 10.1038/s41598-017-01863-5 PMC5431780 28490737

[59] Mizutani N, Nabe T, Yoshino S (2013). Interleukin-33 and alveolar macrophages contribute to the mechanisms underlying the exacerbation of IgE-mediated airway inflammation and remodelling in mice. Immunology.

[60] Préfontaine D (2009). Increased expression of IL-33 in severe asthma: evidence of expression by airway smooth muscle cells. J Immunol.

[61] Wang Y, Wang L, Hua S (2017). Interleukin-33 in children with asthma: a systematic review and meta-analysis. Allergol Immunopathol (Madr).

[62] Fogli LK (2013). T cell-derived IL-17 mediates epithelial changes in the airway and drives pulmonary neutrophilia. J Immunol.

[63] Molet S (2001). IL-17 is increased in asthmatic airways and induces human bronchial fibroblasts to produce cytokines. J Allergy Clin Immunol.

[64] Al-Ramli W (2009). T(H)17-associated cytokines (IL-17A and IL-17F) in severe asthma. J Allergy Clin Immunol.

[65] Sergejeva S, Ivanov S, Lötvall J, Lindén A (2005). Interleukin-17 as a recruitment and survival factor for airway macrophages in allergic airway inflammation. Am J Respir Cell Mol Biol.

[66] Kudo M (2012). IL-17A produced by αβ T cells drives airway hyper-responsiveness in mice and enhances mouse and human airway smooth muscle contraction. Nat Med.

[67] Busse WW (2013). Randomized, double-blind, placebo-controlled study of Brodalumab, a human anti–IL-17 receptor monoclonal antibody, in moderate to severe asthma. Am J Respir Crit Care Med.

[68] Kirsten A (2013). The anti-IL-17A antibody secukinumab does not attenuate ozone-induced airway neutrophilia in healthy volunteers. Eur Respir J.

[69] Clinicaltrials.gov. Available at: https://clinicaltrials.gov/ct2/show/NCT01478360?term=secukinumab&cond=Asthma&rank=1. (Accessed 7 Aug 2018).

[70] Mukaida N (2003). Pathophysiological roles of interleukin-8/CXCL8 in pulmonary diseases. Am J Physiol Lung Cell Mol Physiol.

[71] Chapman RW (2009). CXCR2 antagonists for the treatment of pulmonary disease. Pharmacol Ther.

[72] Chapman RW (2007). A novel, orally active CXCR1/2 receptor antagonist, Sch527123, inhibits neutrophil recruitment, mucus production, and goblet cell hyperplasia in animal models of pulmonary inflammation. J Pharmacol Exp Ther.

[73] Nair P (2012). Safety and efficacy of a CXCR2 antagonist in patients with severe asthma and sputum neutrophils: a randomized, placebo-controlled clinical trial. Clin Exp Allergy.

[74] O'Byrne PM (2016). Efficacy and safety of a CXCR2 antagonist, AZD5069, in patients with uncontrolled persistent asthma: a randomised, double-blind, placebo-controlled trial. Lancet Respir Med.

[75] Webb DC (2000). Integrated signals between IL-13, IL-4, and IL-5 regulate airways Hyperreactivity. J Immunol.

